# Commentary: Serum Calprotectin Is a Valid Biomarker in Distinction of Bacterial Urinary Tract Infection From Viral Respiratory Illness in Children Under 3 Years of Age

**DOI:** 10.3389/fped.2022.921939

**Published:** 2022-06-21

**Authors:** Boris Adasevic, Daniel Turudic, Danko Milosevic

**Affiliations:** ^1^Department of Pediatrics, General Hospital Zabok and Hospital of Croatian Veterans, Bracak, Croatia; ^2^Department of Pediatrics, University Hospital Centre Zagreb, Zagreb, Croatia; ^3^School of Medicine, University of Zagreb, Zagreb, Croatia

**Keywords:** urinary tract infections, respiratory infections, serum calprotectin, biomarkers, children

## Introduction

This article aims to analyze the performance of various biomarkers in distinguishing urinary tract infections (UTI) from viral respiratory illness (RI) using receiver operating characteristic (ROC) and logistic regression (LR) (1). We performed a literature search of three widely available databases (Pubmed, Scopus, Medline), which revealed several similar articles discussing differential diagnoses of UTI from viral RI (2–5).

## Relevant Subsection

Suprapubic aspiration or catheterization is the preferred method of obtaining an adequate urine sample and reliable urine culture when there is a concern for UTI in children who are not toilet trained. Since bag urine specimens have a high contamination rate (up to 63%), the American Academy of Pediatrics does not recommend using this method to obtain a urine culture in such children. Urine culture obtained by this method cannot be considered a “gold standard.” Since the diagnosis of bacterial UTI in the published study is likely inaccurate, the statement of the commented article, with sensitivity and specificity of sCal identifying bacterial UTI from RI were 77.6 and 69.0%, respectively, is based on unreliable data (6–11). Since bag urine specimens were used for urine collection, bacterial UTIs were likely overdiagnosed (1).

White blood cell count (WBC) and absolute neutrophil count (ANC) variables analyzed by ROC analysis and univariate binary LR show a higher area under the curve (AUC) and Youden index (YI) values than serum calprotectin (sCal) (Table 4, Table 7). The 95% confidence interval (CI) for AUC in ROC analysis is missing from Table 4, despite being commented on in Results (page 4), and ROC curves for all tested variables have not been illustrated. sCal specificity commented in Results differs between Table 4 and Figure 2 (sCal specificity in Figure 2 and Results—page 4 is 69% *vs*. 79% in Table 4). Selected results from the predictive model generated by multivariate binary LR were intentionally commented in Results (page 5), but the model itself was not shown. The model should have been presented separately, and variable selection methods (for example forward stepwise, backward stepwise, hierarchical *etc*.) should have been explained step by step. From the model presented in this manner, it is unclear how the variables were selected and excluded within the model. The prediction values between each and every variable were also not included. Other researchers cannot reproduce the model presented in this manner. This shows a *selection bias*.

When comparing multiple tests using ROC analysis, it would be necessary to compare multiple ROC curves in a single plot. A pairwise ROC curve analysis can simultaneously compare several diagnostic tests on the same subjects to consider the covariance and significant differences between ROC curves. There is also a significant age difference between UTI cases and viral RI controls (Table 2, UTI age 3.75 (2.0–6.13) *vs*. 14.0 (5.0–21.25) for viral RI, *p* < 0.0001). Age is a known confounder that can deviate the ROC curve from its true location in ROC space, thus resulting in over- or under-estimation of its accuracy (12). AUC and Youden index are standard measures of how well a parameter can distinguish between two diseases as they give equal weight to false positive and false negative values. Variables with the same values would have the same proportion of total misclassified patients (13–15). ANC and WBC have more potential for distinguishing between these two diseases above other variables, sCal included.

We found published biomarkers differentiating RI from UTI in children that authors neglected to mention or include in the article. Blood myxovirus resistance protein A has better AUC than sCal (0.96, 95% CI 0.89–1.0) (16).

The authors cite an article analyzing UTIcalc, which uses standard parameters for UTI assessment. UTIcalc was developed to estimate the probability of UTI in children. It reduced testing by 8.1% and decreased the number of missed UTIs and treatment delays, with a faster UTI diagnosis. Why have the authors missed the opportunity to compare UTIcalc with sCal in their work (17)?

The authors also commented an article that describes the superior accuracy of procalcitonin (PCT) for UTI diagnosis (18). The group of children in the cited article without verified renal scarring by DMSA had PCT values (median 0.40; 0.10–8.25) resembling children in the commented article (median 0.31; 0.11–2.05). Children with renal scarring had significantly higher PCT values (9.58, CI 4.0–28.68) (18). As the authors did not perform a DMSA scan, PCT values in commented article correspond with non-complicated UTIs without renal scarring. The high cost of PCT testing was also addressed (1). However, the cost of sCal testing in Croatia is about 30% more than PCT testing 1, (Supplementary data, Table 1).

**Table 1:** Supplementary data—cost of various biomarkers in Croatia.

**Test**	**Price (approximately)**
sCal	29.13 €
PCT	20.27 €
CRP	6.62 €
CBC (with ANC, WBC, N%)	6 €

## Discussion

From the article, it can be deduced that the authors have a dilemma in distinguishing UTIs from viral RI. Recognizing bacterial UTI from viral RI belongs to basic medical skills and can be easily distinguished with clinical examination and common laboratory tests.

From the title itself, and the whole structure of the paper, the article particularly advocates the use of sCal in distinguishing UTIs from viral RI. As sCal is not a new biomarker used to detect bacterial infections, it is unclear why these two diseases were particularly selected for analysis, or what exactly applies to the claim that sCal has “substantial added value in the early management of a child with fever and positive urinalysis?” Are we more and more neglecting basic medical skills at the expense of the ever-increasing and exorbitant price of laboratory testing?

sCal use in routine practice for distinguishing UTIs from viral RI is entirely unacceptable, especially for developing countries and could mislead younger pediatricians about its uncontrolled use.

## Conclusion

In most cases, basic medical skills, medical history, clinical examination and follow-up, proper urine sampling and analysis, and simple laboratory tests (WBC, ANC, *etc*.) are sufficient to distinguish between bacterial UTIs and viral RI. Sophisticated and expensive laboratory tests (sCal included) in no way could supersede the basic medical skills and necessary laboratory tests. This should be the real message of the article.

## Author Contributions

BA, DT, and DM contributed to conception and design of the study and revised the sections of the manuscript. DT performed the statistical commentary. BA wrote the first draft of the manuscript. All authors contributed to manuscript revision, read, and approved the submitted version.

## Conflict of Interest

The authors declare that the research was conducted in the absence of any commercial or financial relationships that could be construed as a potential conflict of interest.

## Publisher's Note

All claims expressed in this article are solely those of the authors and do not necessarily represent those of their affiliated organizations, or those of the publisher, the editors and the reviewers. Any product that may be evaluated in this article, or claim that may be made by its manufacturer, is not guaranteed or endorsed by the publisher.
